# Variants in genes related to development of the urinary system are associated with Mayer–Rokitansky–Küster–Hauser syndrome

**DOI:** 10.1186/s40246-022-00385-0

**Published:** 2022-03-31

**Authors:** Chunfang Chu, Lin Li, Shenghui Li, Qi Zhou, Ping Zheng, Yu-Di Zhang, Ai-hong Duan, Dan Lu, Yu-Mei Wu

**Affiliations:** 1 Department of Gynecology, Beijing Obstetrics and Gynecology Hospital, Capital Medical University, Beijing Maternal and Child Health Care Hospital, Chaoyang, Beijing, 100026 China; 2 Central Laboratory, Beijing Obstetrics and Gynecology Hospital, Capital Medical University, Beijing Maternal and Child Health Care Hospital, Dongcheng, Beijing, 100006 China; 3 Department of Gynecological Oncology, Beijing Obstetrics and Gynecology Hospital, Capital Medical University, Beijing Maternal and Child Health Care Hospital, Dongcheng, Beijing, 100006 China

**Keywords:** Mayer–Rokitansky–Küster–Hauser syndrome, Whole-exome sequencing, Variant, TBC1D1

## Abstract

Mayer–Rokitansky–Küster–Hauser (MRKH) syndrome, also known as Müllerian agenesis, is characterized by uterovaginal aplasia in an otherwise phenotypically normal female with a normal 46,XX karyotype. Previous studies have associated sequence variants of *PAX8*, *TBX6*, *GEN1*, *WNT4*, *WNT9B*, *BMP4*, *BMP7*, *HOXA10*, *EMX2*, *LHX1*, *GREB1L*, *LAMC1*, and other genes with MRKH syndrome. The purpose of this study was to identify the novel genetic causes of MRKH syndrome. Ten patients with MRKH syndrome were recruited at Beijing Obstetrics and Gynecology Hospital, Capital Medical University, Beijing, China. Whole-exome sequencing was performed for each patient. Sanger sequencing confirmed the potential causative genetic variants in each patient. In silico analysis and American College of Medical Genetics and Genomics (ACMG) guidelines helped to classify the pathogenicity of each variant. The Robetta online protein structure prediction tool determined whether the variants affected protein structures. Eleven variants were identified in 90% (9/10) of the patients and were considered a molecular genetic diagnosis of MRKH syndrome. These 11 variants were related to nine genes: *TBC1D1*, *KMT2D*, *HOXD3*, *DLG5*, *GLI3*, *HIRA*, *GATA3*, *LIFR*, and *CLIP1*. Sequence variants of *TBC1D1* were found in two unrelated patients. All variants were heterozygous. These changes included one frameshift variant, one stop-codon variant, and nine missense variants. All identified variants were absent or rare in gnomAD East Asian populations. Two of the 11 variants (18.2%) were classified as pathogenic according to the ACMG guidelines, and the remaining nine (81.8%) were classified as variants of uncertain significance. Robetta online protein structure prediction analysis suggested that missense variants in TBC1D1 (p.E357Q), HOXD3 (p.P192R), and GLI3 (p.L299V) proteins caused significant structural changes compared to those in wild-type proteins, which in turn may lead to changes in protein function. This study identified many novel genes, especially *TBC1D1*, related to the pathogenesis of MRKH syndrome. The identification of these variants provides new insights into the etiology of MRKH syndrome and a new molecular genetic reference for the development of the reproductive tract.

**Supplementary Information:**

The online version contains supplementary material available at 10.1186/s40246-022-00385-0.

## Introduction

Mayer–Rokitansky–Küster–Hauser (MRKH) syndrome (MIM: %277,000), also known as Müllerian agenesis [1], Müllerian aplasia, or Müllerian dysgenesis or congenital absence of the uterus and vagina, is characterized by uterovaginal aplasia in an otherwise phenotypically normal female with a normal 46,XX karyotype. MRKH syndrome affects approximately one in 5000 live female births [2], and has been reported in approximately 16% of patients with primary amenorrhea [3]. When only the reproductive organs (uterus, fallopian tubes, cervix, and the upper part of the vagina) are affected, this condition is classified as MRKH syndrome type I. Some women with MRKH syndrome also have abnormalities in other organs of the body; in these cases, the disease is classified as MRKH syndrome type II [4]. Affected individuals often display renal, skeletal, and heart defects and hearing loss [5].

MRKH syndrome is directly caused by incomplete development of the Müllerian ducts. Genetic and/or environmental factors that control the formation and morphogenesis of Müllerian ducts are closely related to the MRKH syndrome. During human embryonic development, Müllerian ducts form just lateral to the Wolffian duct. Both Müllerian and Wolffian ducts develop from the intermediate mesoderm and on the surface of the mesonephric kidneys. Therefore, MRKH syndrome is usually associated with abnormalities of the renal and axial skeletal systems.

Previous studies have found that genetic perturbations can lead to MRKH syndrome. For example, *PAX8*, *TBX6*, *GEN1*, *WNT4*, *WNT9B*, *BMP4*, *BMP7*, *HOXA10*, *EMX2*, *LHX1*, *GREB1L*, *LAMC1*, and other genes are associated with MRKH syndrome [6–16]. Although these genetic factors have been related to the pathogenesis of MRKH syndrome, the genetic inheritance pattern of MRKH syndrome is very complex, including autosomal dominant, autosomal recessive, digenic, oligogenic, and incomplete penetrance. Although some genetic pathogenic factors have been identified, more unknown factors have not been identified. The identification of additional pathogenic genes will require large-scale genetic research studies at various institutions in different countries.

In this study, we aimed to explore the novel genetic causes of MRKH syndrome using whole-exome sequencing (WES) technology. We recruited 10 patients with MRKH syndrome and performed WES and family genetic analysis on them. We attempted to identify novel genetic pathogenic factors associated with MRKH syndrome.

## Materials and methods

### Patients

Ten patients diagnosed with MRKH syndrome with a 46,XX karyotype were recruited at Beijing Obstetrics and Gynecology Hospital from January 2019 to May 2021. The clinical conditions and manifestations of the ten patients are presented in Table 1. Five milliliters of peripheral blood were collected from each patient for further genetic analysis.

**Table 1:** Clinical information of the MRKH patients

Patient No	Age at diagnosis	ESHRE and ESGE classification 2021	ASRM Müllerian anomalies	Uterus	Ovaries	Urinary system	Symptoms	Associated malformations	Classification
Fc-M-1	6	U5bC4V4	Müllerian agenesis	Full uterine aplasia	Normal	The left pelvic kidney without function and the right kidney compensatory enlarged	No symptoms, Ultrasound found	Congenital anal atresia with vestibular fistula; Ventricular septal defect; accessory auricle	Type II
Fc-M-2	15	U5bC4V4	Müllerian agenesis	Bilateral uterine remnants without rudimentary cavity	Normal	Normal	Primary amenorrhea	ND	Type I
Fc-M-3	17	U5bC4V4	Müllerian agenesis	Bilateral uterine remnants without rudimentary cavity	Normal	Normal	Primary amenorrhea and dyspareunia	ND	Type I
Fc-M-4	16	U5bC4V4	Müllerian agenesis	Full uterine aplasia	Normal	Normal	Primary amenorrhea and dyspareunia	ND	Type I
Fc-M-5	17	U5bC4V4	Müllerian agenesis	Bilateral uterine remnants without rudimentary cavity	Normal	Normal	Primary amenorrhea and dyspareunia	ND	Type I
Fc-M-6	18	U5bC4V4	Müllerian agenesis	Bilateral uterine remnants without rudimentary cavity	Normal	Normal	Primary amenorrhea and dyspareunia	ND	Type I
Fc-M-7	18	U5bC4V4	Müllerian agenesis	Bilateral uterine remnants without rudimentary cavity	Normal	Normal	Primary amenorrhea and dyspareunia	Congenital cleft palate; bilateral fallopian tubal dysplasia	Type II
Fc-M-8	16	U5bC4V4	Müllerian agenesis	Bilateral uterine remnants without rudimentary cavity	Normal	Normal	Primary amenorrhea and dyspareunia	ND	Type I
Fc-M-9	16	U5bC4V4	Müllerian agenesis	Full uterine aplasia	Normal	Normal	Primary amenorrhea and dyspareunia	dextroversion of the heart; persistent left superior vena cava	Type II
Fc-M-10	15	U5bC4V4	Müllerian agenesis	Bilateral uterine remnants without rudimentary cavity	Normal	Normal	Primary amenorrhea and dyspareunia	ND	Type I

ESHRE, European Society of Human Reproduction and Embryology; ESGE, European Society of Gastrointestinal Endoscopy; ASRM, American Society for Reproductive Medicine

Classification of the MRKH syndrome: Type I MRKH syndrome refers to isolated uterovaginal agenesis with no associated extragenital malformations, Type II refers to all cases with any associated extragenital abnormality (renal, skeletal, and others)

All patients reported no family history of genital and other organ malformations

ND, not described

### WES analysis

Genomic DNA from each patient was extracted from the peripheral blood using the QIAamp DNA Blood Kit (Qiagen, Valencia, CA, USA). WES was performed as previously described [17]. The functional effects of the variants (damaging or not) were predicted using the PolyPhen-2, SIFT, MutationTaster, LRT, and FATHMM-MKL algorithms. The desired variants were filtered using two criteria: (i) missense, nonsense, frameshift, or splice site variants; and (ii) variants with minor allele frequency < 1%. The minor allele frequency information was obtained by referring to the Genome Aggregation Database (gnomAD, http://gnomad.broadinstitute.org/), 1000 Genomes Project (1000G, http://browser.1000genomes.org/index.html), NHLBI Exome Sequencing Project (ESP6500), and our in-house database.

### Sanger sequencing analysis

Sanger sequencing was performed to validate the identified variants and determine if each variant was inherited from a parent. The primer pairs for each gene are listed in Additional file 1: Table S1. Forward or reverse primers were used to sequence the PCR products. Sequencing was performed using an ABI 3730 automatic sequencer (Applied Biosystems, Foster City, CA, USA).

### Protein structure prediction

The three-dimensional structures of wild-type and mutant proteins were predicted using the Robetta online protein structure prediction server (https://robetta.bakerlab.org/) [18]. This tool can predict the three-dimensional structure of a given amino acid sequence. Protein structure alignment was performed using Visual Molecular Dynamics 1.9.3 software (https://www.ks.uiuc.edu/Research/vmd/).

## Results

### WES analysis

Of the 10 women with MRKH syndrome, seven had type I MRKH syndrome and the other three had type II MRKH syndrome (Table 1). WES helped to identify 11 variants in 90% (9/10) of the patients and was considered a molecular genetic diagnostic tool of MRKH syndrome. These 11 variants involved nine genes: *TBC1D1* (Fc-M-1 and Fc-M-3), *KMT2D* (Fc-M-2 and Fc-M-7), *LIFR* (Fc-M-2), *HOXD3* (Fc-M-4), *DLG5* (Fc-M-6), *CLIP1* (Fc-M-7), *GLI3* (Fc-M-8), *HIRA* (Fc-M-9), and *GATA3* (Fc-M-10) (Table 2). All the variants were heterozygous. These changes included one frameshift variant, one stop-codon variant, and nine missense variants (Table 2). All the identified variants were absent or very rare in the gnomAD East Asian population (Table 2). Two of the 11 variants (18.2%) were classified as pathogenic variants according to the American College of Medical Genetics and Genomics (ACMG) guidelines. The remaining nine variants (81.8%) were classified as variants of uncertain significance (VUS).

**Table 2:** In silico analysis of sequence variants found by WES in MRKH patients

Case ID	Zygosity	Gene	Ref mRNA No	Mutation type	Variants	Amino acid change	GnomAD-EAS	PolyPhen2/SIFT/MutationTaster/LRT/FATHMM-MKL	ACMG
Fc-M-1	Hetero	*TBC1D1*	NM_015173	frameshift	c.2553delC	p.R854Efs*24	0	NA/NA/D/NA/NA	P: PVS1 + PM2 + PP3
Fc-M-2	Hetero	*KMT2D*	NM_003482	missense	c.2992C>G	p.P998A	0.0000557	B/D/N/N/D	VUS: BP4
Fc-M-2	Hetero	*LIFR*	NM_002310	missense	c.1418C>G	p.S473C	0	P/D/N/N/N	VUS: PM2 + BP4
Fc-M-3	Hetero	*TBC1D1*	NM_015173	missense	c.1069G>C	p.E357Q	0.0002	D/D/D/D/D	VUS: PM2 + PP3 + BP1
Fc-M-4	Hetero	*HOXD3*	NM_006898	missense	c.575C>G	p.P192R	0	D/D/D/D/D	VUS: PM2 + PP3
Fc-M-6	Hetero	*DLG5*	NM_004747	stop codon	c.418C>T	p.Q140*	0	NA/NA/D/N/D	P: PVS1 + PM2 + PP3
Fc-M-7	Hetero	*KMT2D*	NM_003482	missense	c.1754C>T	p.P585L	0	D/D/D/NA/D	VUS: PM2 + BP4
Fc-M-7	Hetero	*CLIP1*	NM_002956	missense	c.1498C>T	p.R500C	0	D/D/D/N/D	VUS: PM2 + PP3
Fc-M-8	Hetero	*GLI3*	NM_000168	missense	c.895C>G	p.L299V	0	P/D/D/D/D	VUS: PM2 + PP3 + BP1
Fc-M-9	Hetero	*HIRA*	NM_003325	missense	c.845A>G	p.K282R	0	B/T/D/D/D	VUS: PM2 + BP1
Fc-M-10	Hetero	*GATA3*	NM_002051	missense	c.1178C>T	p.P393L	0.00005631	B/D/D/D/D	VUS: PM2 + PP2 + PP3

The Genome Aggregation Database (gnomAD) is a resource developed by an international coalition of investigators, with the goal of aggregating and harmonizing both exome and genome sequencing data from a wide variety of large-scale sequencing projects. In this study, we referred to the allele frequencies in the East Asian (EAS) population

Pathogenicity items: Polyphen2: D, probably damaging; P, possibly damaging; B, benign. SIFT: D, damaging; T, tolerated. MutationTaster: D, disease causing; N, polymorphism. LRT: D, deleterious; N, neutral. FATHMM-MKL: D, damaging; N, neutral. NA, not applicable

ACMG item: ACMG, American College of Medical Genetics and Genomics guidelines; VUS, variant of uncertain significance; P, pathogenic variant; LB, likely benign variant

### Novel candidate genes of MRKH syndrome

#### TBC1D1

**Fig. 1:**
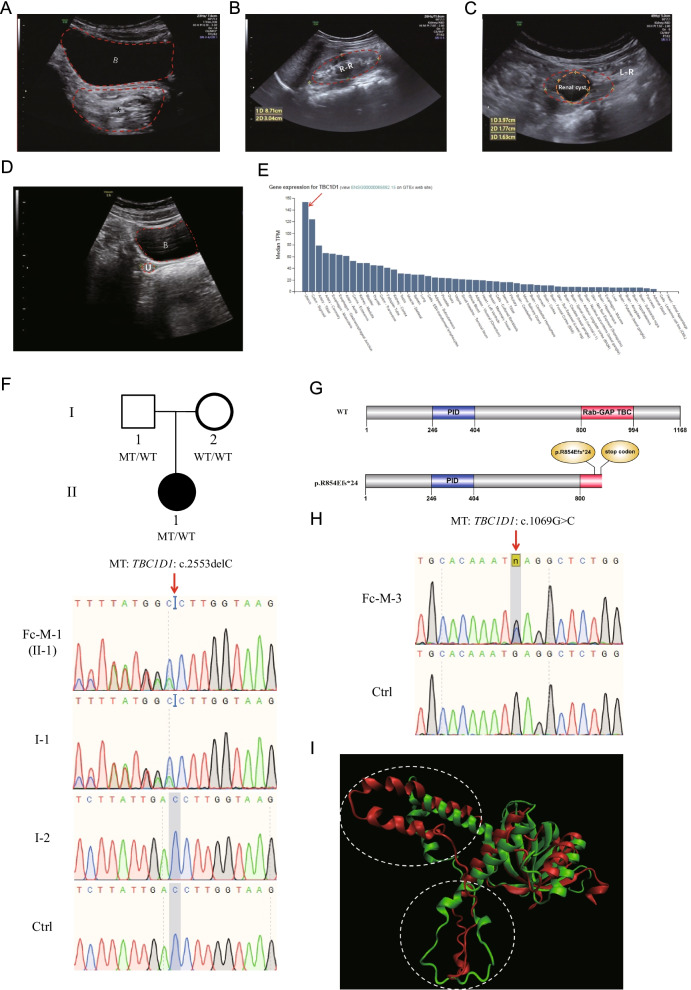
*TBC1D1* mutation in patients with MRKH. **A** Image of patient Fc-M-1 with a diagnosis of MRKH. No uterine echo was evident in the pelvic ultrasound (indicated by *) behind the bladder (denoted by **B**). **B** Image of patient Fc-M-1 with a diagnosis of MRKH. The right renal (R–R) region is enlarged. **C** Image of patient Fc-M-1 with a diagnosis of MRKH. The left renal (L–R) region was dysplastic and located in the left lower abdomen (pelvic ectopic kidney). The region was 4.0 cm in length. A cystic cavity measuring 1.8 * 1.6 cm was evident. The renogram showed that the left kidney had no function. **D** Pelvic ultrasound image of patient Fc-M-3 with a diagnosis of MRKH. B denotes bladder and U denotes aplastic uterus without rudimentary cavity. **E** *TBC1D1* is highly expressed in the human uterus. The data were obtained from an online database (https://varsome.com/gene/TBC1D1). The red arrow denotes the expression level of *TBC1D1* in the human uterus. **F** Sanger sequencing confirmation of the heterozygous *TBC1D1* variant in patient Fc-M-1. The patient’s father (I-1) also carried the same heterozygous variant. The patient’s mother (I-2) harbored two wild-type (WT) alleles. The red arrow indicates the variant site (c.2553delC); MT, mutated allele. **G** The domain and mutation in TBC1D1. Full-length TBC1D1 is 1168 amino acids (aa), and includes the PID domain from aa 246 to 404 (blue box) and the catalytic Rab-GAP TBC domain from aa 800 to 994 (red box). The p.R854Efs*24 mutation results in a predicted 23-aa frameshift sequence in the protein resulting in a nonsense mutation; WT, wild type allele. (H) Sanger sequencing validating the *TBC1D1* variant in patient Fc-M-3. The red arrow indicates the variant site (c.1069G>C). **I** The wild-type (green) and p.E357Q mutant protein (red) structure for amino acids at positions 164 to 371 were predicted by RoseTTAFold. The wild-type sequence and the p.E357Q mutant sequence were aligned by VMD software

We identified *TBC1D1* variants in two unrelated patients, Fc-M-1 and Fc-M-3 (Table 2). Fc-M-1 was diagnosed as type II MRKH syndrome (European Society of Human Reproduction and Embryology [ESHRE] classification: U5bC4V4) with full uterine agenesis, left pelvic kidney dysfunction, congenital anal atresia with vestibular fistula, ventricular septal defect, and accessory auricle (Table 1, Fig. 1A–C). Fc-M-3 was diagnosed as type I MRKH syndrome (ESHRE classification: U5bC4V4) with uterine remnants without a rudimentary cavity (Table 1 and Fig. 1D).

*TBC1D1* was highly expressed in the uterus (Fig. 1E). Fc-M-1 harbored a frameshift c.2553delC (p.R854Efs*24) variant (Fig. 1F) inherited from her father (Fig. 1F). Therefore, *TBC1D1* variant c.2553delC was associated with MRKH syndrome within this family. The c.2553delC variant of *TBC1D1* was predicted to produce a truncated p.R854Efs*24 protein, which would destroy the Rab-GAP TBC domain of the TBC1D1 protein and lead to the loss of C-terminal sequences (Fig. 1G). The c.2553delC variant was absent in the gnomAD East Asian population. It was classified as a pathogenic variant (PVS1 + PM2 + PP3) according to ACMG guidelines.

Fc-M-3 carried a missense c.1069G>C (p.E357Q) variant confirmed by Sanger sequencing (Fig. 1H). Due to the unavailability of samples from the patient’s mother and father, we could not determine how this variant was transmitted. The allele frequency of the c.1069G>C variant in the gnomAD East Asian population was 0.0002. The variant was predicted to be a damaging variant by all five algorithms we used and was classified as VUS (PM2 + PP3 + BP1) according to the ACMG guidelines (Table 2). p.E357Q was located in a PTB_TBC1D1_like domain (TBC1 domain family member 1 and related protein phosphotyrosine binding (PTB)), which contains amino acids at positions 164 to 371. Wild-type (WT) and mutant protein structures for amino acids at positions 164 to 371 were predicted by the Robetta fold. The domain structural prediction results revealed significant structural changes in at least two regions between the protein carrying the p.E357Q variant and the WT protein (Fig. 1I), suggesting that the p.E357Q variant might affect the function of TBC1D1.

#### DLG5

**Fig. 2:**
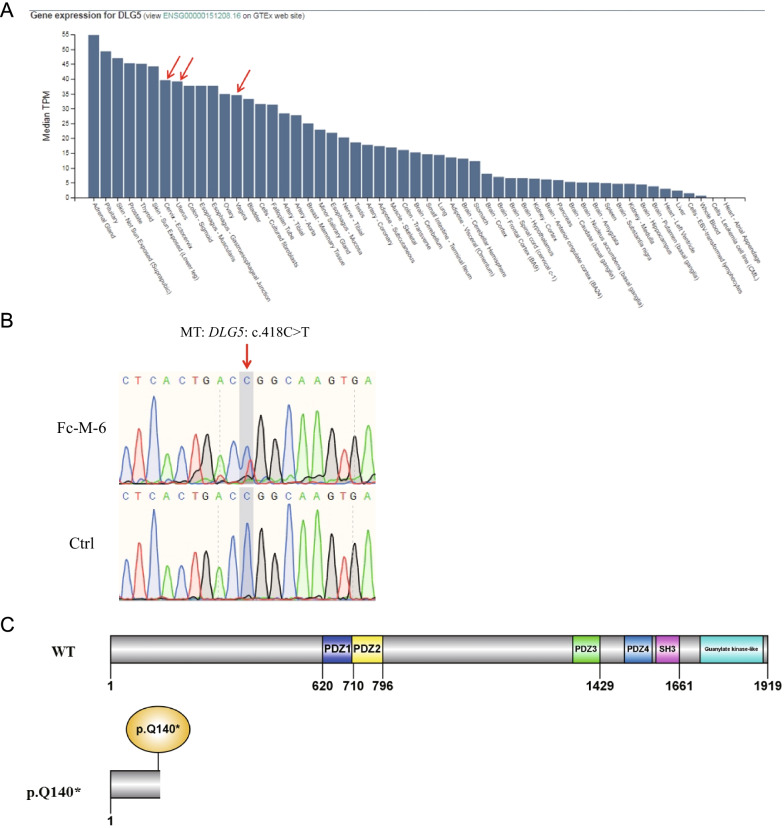
*DLG5* variant is associated with MRKH syndrome. **A** *DLG5* is highly expressed in the human cervix, uterus, and vagina. The data were obtained from an online database (https://varsome.com/gene/DLG5). The red arrows denote the expression level of *DLG5* in the human cervix, uterus, and vagina. **B** Sanger sequencing validating the *DLG5* variant in patient Fc-M-6. The red arrow indicates the variant site c.418C>T. **C** The domain and mutation in DLG5. Full-length DLG5 is 1919 amino acids long. The Q140* mutation resulted in a nonsense mutation, which lost nearly all of the functional domains; WT, wild type allele

We identified a *DLG5* variant in patient Fc-M-6 who was diagnosed as having type I MRKH syndrome (ESHRE classification: U5bC4V4) with primary amenorrhea and dyspareunia (Table 1). *DLG5* was highly expressed in the human cervix, uterus, and vagina (Fig. 2A). Fc-M-6 harbored the *DLG5* stop-codon gained variant c.418C>T (p.Q140*) (Table 2). This variant was confirmed by Sanger sequencing (Fig. 2B) and classified as a pathogenic variant (PVS1 + PM2 + PP3) according to the ACMG guidelines (Table 2). The c.418C>T variant of *DLG5* was predicted to produce a truncated p.Q140* protein, which would destroy all functional domains in the DLG5 protein (Fig. 2C).

#### HOXD3

**Fig. 3:**
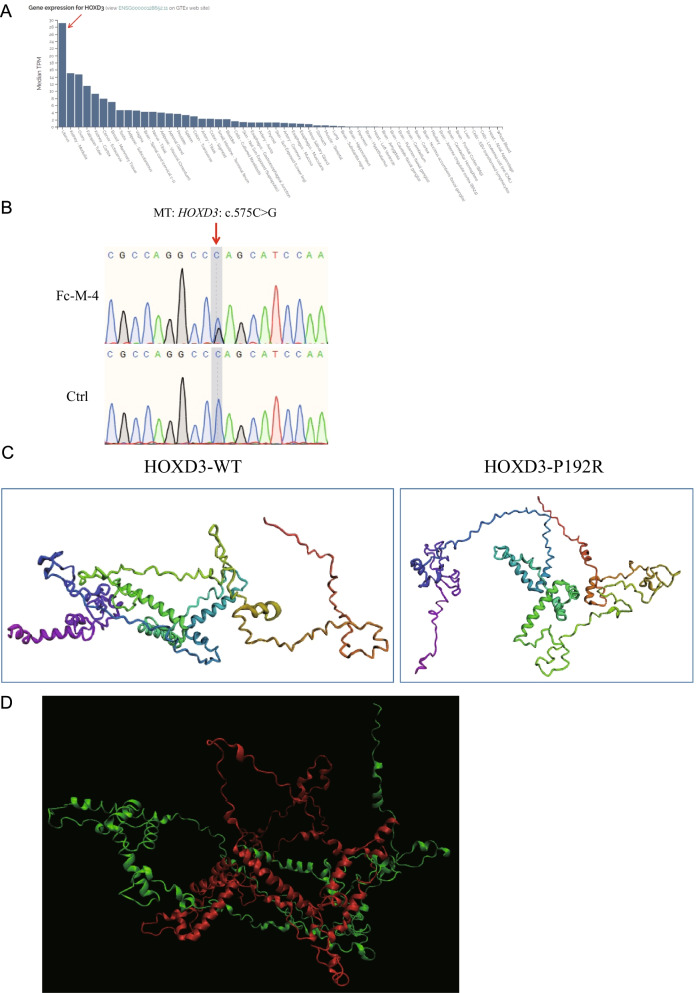
*HOXD3* variant is associated with MRKH syndrome. **A** *HOXD3* is highly expressed in the human uterus. The data were obtained from an online database (https://varsome.com/gene/HOXD3). The red arrow denotes the expression level of *HOXD3* in the human uterus. **B** Sanger sequencing validating the *HOXD3* variant in patient Fc-M-4. The red arrow indicates the variant site c.575C>G. **C** The full-length wild-type (WT) HOXD3 protein and P192R mutant protein structures were predicted by RoseTTAFold. **D** The predicted protein structures for the WT HOXD3 protein (green) and the P192R mutant protein (red) were aligned

We identified a *HOXD3* missense variant in patient Fc-M-4, who was diagnosed with type I MRKH syndrome (ESHRE classification: U5bC4V4) with primary amenorrhea and dyspareunia (Table 1). *HOXD3* was highly expressed in the human uterus (Fig. 3A). Fc-M-4 carried a heterozygous *HOXD3* variant, c.575C>G (p.P192R) (Table 2), which was confirmed by Sanger sequencing (Fig. 3B) and classified as a VUS variant (PM2 + PP3) according to the ACMG guidelines (Table 2). The structural prediction results revealed pronounced structural changes between the HOXD3-WT protein and the mutant protein (p.P192R) (Fig. 3C). Alignment of the two proteins was difficult (Fig. 3D).

#### GLI3

**Fig. 4:**
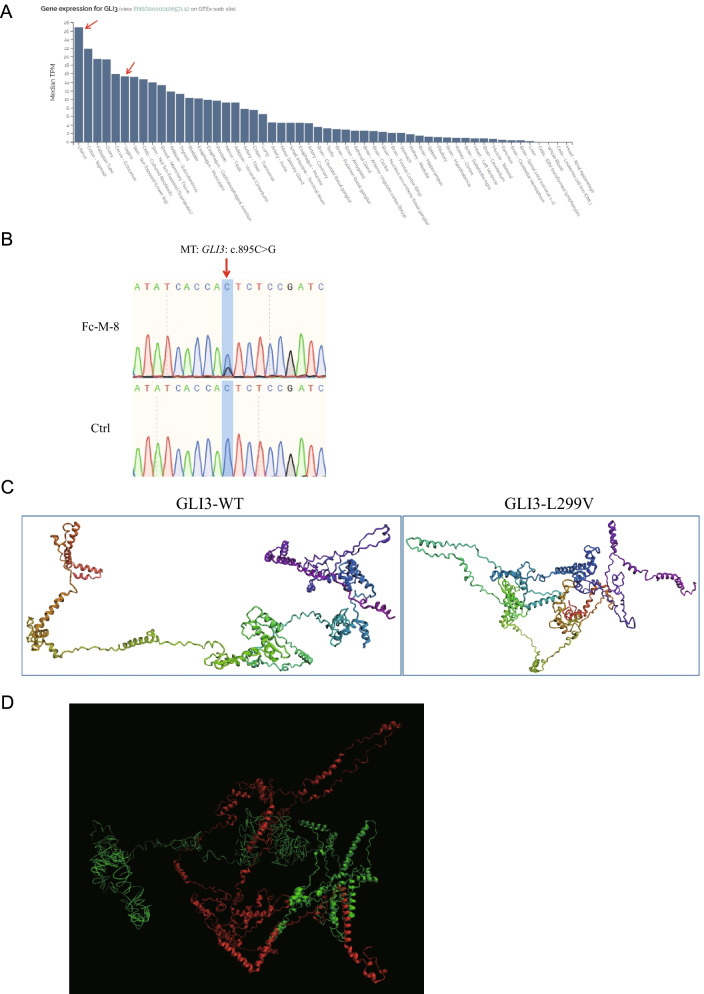
*GLI3* variant is associated with MRKH syndrome. **A** *GLI3* is highly expressed in the human uterus and vagina. The data were obtained from an online database (https://varsome.com/gene/GLI3). The red arrows denote the expression level of *GLI3* in the human uterus and vagina. **B** Sanger sequencing validating the *GLI3* variant in patient Fc-M-8. The red arrow indicates the variant site c.895C>G. **C** The 1–683 amino acid sequence for wild-type (WT) GLI3 protein and L299V mutant protein structures were predicted by RoseTTAFold. **D** The predicted protein sequence (1–683 amino acids) structures for WT GLI3 protein (green) and the L299V mutant protein (red) were aligned

We also identified a *GLI3* missense variant in patient Fc-M-8, who was diagnosed with type I MRKH syndrome (ESHRE classification: U5bC4V4) with primary amenorrhea and dyspareunia (Table 1). *GLI3* was highly expressed in the human uterus and vagina (Fig. 4A). Fc-M-8 carried a heterozygous *GLI3* variant c.895C>G (p.L299V) (Table 2), which was confirmed by Sanger sequencing (Fig. 4B) and classified as a VUS variant (PM2 + PP3 + BP1) according to the ACMG guidelines (Table 2). The structural prediction results showed that there were significant structural changes between the GLI3-WT protein and the mutant protein (p.L299V) (Fig. 4C). Alignment of the GLI3-WT and GLI3-mutant proteins was difficult (Fig. 4D).

#### Other genes associated with MRKH syndrome

We also identified variants of *KMT2D*, *LIFR*, *CLIP1*, *HIRA*, and *GATA3*. All variants were classified as VUS according to the ACMG guidelines (Table 2). All variants were confirmed using Sanger sequencing (Additional files 3 and 4: Figure S2A and S3A; some data not shown). Some of the mutant proteins that harbored the variants were analyzed by protein structure prediction (Additional files 3 and 4: Figure S2B, C, S3B, and C).

Two variants of *KMT2D* (c.2992C>G; P998A, and c.1754C>T; P585L) were found in two unrelated patients. The P998A variant was found in patient Fc-M-2 (Table 2), who was diagnosed with type I MRKH syndrome with primary amenorrhea and bilateral uterine remnants, without a rudimentary cavity (Table 1). The P585L variant was carried by patient Fc-M-7 (Table 2), who was diagnosed with type II MRKH syndrome with bilateral uterine remnants without a rudimentary cavity, congenital cleft palate, and bilateral fallopian tubal dysplasia (Table 1).

## Discussion

Using WES and genetic analysis, this study identified several novel genetic variants that may lead to MRKH syndrome. Next, we discuss these novel genes involved in the pathogenesis of MRKH syndrome.

### TBC1D1

*TBC1D1* encodes a Rab-GTPase-activating protein and is involved in regulating the trafficking of GLUT4 storage vesicles to the cell surface [19]. Previous studies have found that heterozygous mutation of *TBC1D1* is associated with congenital anomalies of the kidneys and urinary tract (CAKUT) [20, 21]. The *TBC1D1* mutation may promote the pathogenesis of CAKUT through its role in glucose homeostasis [20]. Patient Fc-M-1 harboring the *TBC1D1* truncating variant found in this study also had CAKUT; the left pelvic kidney did not function and the right kidney was enlarged as a compensatory response. Type II MRKH syndrome is usually complicated by abnormalities in the urinary system. Therefore, the study findings suggest that attention should be paid to whether patients with type II MRKH syndrome, especially those with urinary system abnormalities, carry genetic variants related to CAKUT. A previous study by our group has also shown that sequence variants related to CAKUT could be associated with another complex reproductive tract malformation, which is related to the Herlyn–Werner–Wunderlich syndrome [17].

### DLG5

*Dlg5* is required for epithelial tube maintenance in the mouse brain and kidney. *Dlg5* gene knockout mice exhibit hydrocephalus and renal cysts [22]. Heterozygous sequence variants of *DLG5* are associated with ureteropelvic junction obstruction or renal agenesis [23]. Gene expression data of *DLG5* in humans in the present study (Fig. 2A) showed that *DLG5* was highly expressed in the tissues of the reproductive tract, including the cervix, uterus, and vagina. Therefore, the DLG5 protein may play an important role in the development of the reproductive tract. In this study, patient Fc-M-6 with MRKH syndrome harbored a rare *DLG5* truncating variant, Q140* (Fig. 2C), which is classified as a pathogenic variant according to ACMG guidelines. Q140* lacks almost all functional domains of the DLG5 protein, so the variant may lead to the loss of function of the protein. As few patients with MRKH syndrome were included in this study, only one *DLG5* variant was found. We expect our follow-up research or other research groups to identify *DLG5* variants in unrelated patients with MRKH syndrome and provide more evidence of the association of mutations in this gene with MRKH syndrome.

### KMT2D

Previous studies have reported that *KMT2D* mutations can lead to Kabuki syndrome [24, 25]. *KMT2D* gene variants are also related to CAKUT and renal agenesis [26–29]. In the present study, the Fc-M-2 and Fc-M-7 patients harbored a *KMT2D* variant. Both patients also carried another genetic variant. Fc-M-2 carried a variant of the *LIFR* gene (Table 2), which is related to the pathogenesis of CAKUT [30]. Fc-M-7 harbored a variant in the *CLIP1* gene (Table 2), which is also a candidate gene for CAKUT [29]. The findings suggest that Fc-M-2 and Fc-M-7 are related to digenic inheritance. *KMT2D*, *LIFR*, and *CLIP1* are associated with CAKUT, which also indicates that perturbations of renal development-related genes may affect the normal development of reproductive tracts, including the uterus and vagina [17, 31].

### Other genes

We also found several genes, including *HOXD3*, *GLI3*, *HIRA*, and *GATA3*, which may be associated with MRKH syndrome. *HOXD3* is predominantly expressed in the uterus and kidney (https://varsome.com/gene/HOXD3), suggesting its important roles in reproductive and urinary tract development. *GLI3* is highly expressed in the uterus (https://varsome.com/gene/GLI3). Variants in *GLI3 *have also been associated with CAKUT or renal agenesis [23, 26, 32]. *HIRA* encodes a histone chaperone and is considered the primary candidate gene in DiGeorge syndrome. Deletion or duplication in chromosomal loci 22q11.21 containing the *HIRA* gene has been associated with MRKH syndrome [33, 34]. *GATA3* is expressed in Wolffian ducts at the time of their emergence in the embryonic intermediate mesoderm [35]. GATA3 mutations cause hypoparathyroidism, deafness, renal dysplasia syndrome, and CAKUT [26, 36, 37]. Female genital tract malformations, such as uterus didelphys with septate vagina and septate uterus, have also been observed in patients with hypoparathyroidism, deafness, and renal dysplasia syndrome harboring *GATA3* mutations ^38^. Considering the close developmental relationship of the Müllerian and Wolffian ducts, mutations in the *HOXD3*, *GLI3*, *HIRA*, and *GATA3* genes might lead to the abnormal fusion of the caudal parts of the Müllerian ducts needed for the normal development of the uterus and vagina, resulting in MRKH syndrome.

The limitations of this study lie in the following two aspects. First, the sample size of this study is relatively small. MRKH syndrome is a rare disease with an incidence of 1/5000. Patients who come to our hospital for treatment of this disease are very few, so the number of patients who can be recruited in the group is even less. Although the sample size is relatively small, the researchers in our team try their best to find the genetic pathogenic factors of each patient. More patients will be recruited in the future, and we will also focus on whether the pathogenic genes we have found are mutated in the patients enrolled in the future. Secondly, most of these variants found in this study are VUS variants. The reason why this kind of variant is VUS is mainly because the variants have not been rigorously analyzed by functional experiments. We will also continue to study the variants of interest to clarify the molecular mechanism of their pathogenesis.

## Conclusion

Genetic variants, especially in the *TBC1D1* gene, are related to the pathogenesis of MRKH syndrome. This study provides new insights into the etiology of MRKH syndrome and the data are a new molecular genetic reference for the development of the reproductive tract.

## Supplementary Information

**Additional file 1: Table 1.** PCR Primers used in Sanger sequencing validation.

**Additional file 2: Fig. 1** Urinary ultrasound images of patient Fc-M-3. The left renal region (A) and right renal region (B) are normal.

**Additional file 3: Fig. 2** *HIRA* variant is associated with MRKH syndrome. (A) Sanger sequencing validating the *HIRA* variant in patient Fc-M-9. The red arrow indicates the variant site c.845A>G. The patient’s mother did not harbor the variant. (B) The full-length wild-type (WT) HIRA protein and K282R mutant protein structures were predicted by RoseTTAFold. (C) The predicted protein structures for HIRA WT protein (green) and the K282R mutant protein (red) were aligned. The structure and conformation of K282R mutant protein have changed, so it is difficult for the K282R mutant protein to overlap with the WT protein.

**Additional file 4: Fig. 3** *GATA3* variant is associated with MRKH syndrome. (A) Sanger sequencing validating the *GATA3* variant in patient Fc-M-10. The red arrow indicates the variant site c.1178C>T. (B) The full-length GATA3 wild-type (WT) protein and P393L mutant protein structures were predicted by RoseTTAFold. (C) The predicted protein structures for GATA3 WT protein (green) and the P393L mutant protein (red) were aligned. The predicted WT protein structure is more stretched, while the predicted P393L mutant protein structure is more compact. It is difficult for them to structurally overlap.

## Data Availability

The data analyzed in this study are available from the corresponding author upon reasonable request.

## Abbreviations

MRKH: Mayer–Rokitansky–Küster–Hauser
WES: Whole-exome sequencing
ACMG: American College of Medical Genetics and Genomics
gnomAD: Genome Aggregation Database
1000G: 1000 Genomes Project
ESP6500: NHLBI Exome Sequencing Project
VUS: Variants of uncertain significance
ESHRE: European Society of Human Reproduction and Embryology
PTB: Phosphotyrosine binding
CAKUT: Congenital anomalies of the kidney and urinary tract

## References

[1] Pfeifer SM, Attaran M, Goldstein J (2021). ASRM müllerian anomalies classification 2021. Fertil Steril.

[2] Herlin M, Bjørn AM, Rasmussen M, Trolle B, Petersen MB (2016). Prevalence and patient characteristics of Mayer-Rokitansky-Küster-Hauser syndrome: a nationwide registry-based study. Hum Reprod.

[3] Timmreck LS, Reindollar RH (2003). Contemporary issues in primary amenorrhea. Obstet Gynecol Clin North Am.

[4] Grimbizis GF, Gordts S, Di Spiezio SA (2013). The ESHRE/ESGE consensus on the classification of female genital tract congenital anomalies. Hum Reprod.

[5] Morcel K, Camborieux L, Guerrier D (2007). Mayer-Rokitansky-Küster-Hauser (MRKH) syndrome. Orphanet J Rare Dis.

[6] Fontana L, Gentilin B, Fedele L, Gervasini C, Miozzo M (2017). Genetics of Mayer-Rokitansky-Kuster-Hauser (MRKH) syndrome. Clin Genet.

[7] Waschk DE, Tewes AC, Romer T (2016). Mutations in WNT9B are associated with Mayer-Rokitansky-Kuster-Hauser syndrome. Clin Genet.

[8] Sandbacka M, Laivuori H, Freitas E (2013). TBX6, LHX1 and copy number variations in the complex genetics of Mullerian aplasia. Orphanet J Rare Dis.

[9] Tewes AC, Rall KK, Romer T (2015). Variations in RBM8A and TBX6 are associated with disorders of the mullerian ducts. Fertil Steril.

[10] Ledig S, Brucker S, Barresi G, Schomburg J, Rall K, Wieacker P (2012). Frame shift mutation of LHX1 is associated with Mayer-Rokitansky-Kuster-Hauser (MRKH) syndrome. Hum Reprod.

[11] Ekici AB, Strissel PL, Oppelt PG (2013). HOXA10 and HOXA13 sequence variations in human female genital malformations including congenital absence of the uterus and vagina. Gene.

[12] Chen N, Zhao S, Jolly A (2021). Perturbations of genes essential for Müllerian duct and Wölffian duct development in Mayer-Rokitansky-Küster-Hauser syndrome. Am J Hum Genet.

[13] Mikhael S, Dugar S, Morton M (2021). Genetics of agenesis/hypoplasia of the uterus and vagina: narrowing down the number of candidate genes for Mayer-Rokitansky-Küster-Hauser Syndrome. Hum Genet.

[14] Wang L, Zhang Y, Fu X (2020). Joint utilization of genetic analysis and semi-cloning technology reveals a digenic etiology of Müllerian anomalies. Cell Res.

[15] Jacquinet A, Boujemla B, Fasquelle C (2020). GREB1L variants in familial and sporadic hereditary urogenital adysplasia and Mayer-Rokitansky-Kuster-Hauser syndrome. Clin Genet.

[16] Herlin MK, Le VQ, Højland AT (2019). Whole-exome sequencing identifies a GREB1L variant in a three-generation family with Müllerian and renal agenesis: a novel candidate gene in Mayer-Rokitansky-Küster-Hauser (MRKH) syndrome. A case report. Hum Reprod.

[17] Li L, Chu C, Li S (2021). Renal agenesis-related genes are associated with Herlyn-Werner-Wunderlich syndrome. Fertil Steril.

[18] Baek M, DiMaio F, Anishchenko I (2021). Accurate prediction of protein structures and interactions using a three-track neural network. Science.

[19] Roach WG, Chavez JA, Mîinea CP, Lienhard GE (2007). Substrate specificity and effect on GLUT4 translocation of the Rab GTPase-activating protein Tbc1d1. Biochem J.

[20] Kosfeld A, Kreuzer M, Daniel C (2016). Whole-exome sequencing identifies mutations of TBC1D1 encoding a Rab-GTPase-activating protein in patients with congenital anomalies of the kidneys and urinary tract (CAKUT). Hum Genet.

[21] Heidet L, Morinière V, Henry C (2017). Targeted exome sequencing identifies PBX1 as involved in monogenic congenital anomalies of the kidney and urinary tract. J Am Soc Nephrol.

[22] Nechiporuk T, Fernandez TE, Vasioukhin V (2007). Failure of epithelial tube maintenance causes hydrocephalus and renal cysts in Dlg5−/− mice. Dev Cell.

[23] Nicolaou N, Pulit SL, Nijman IJ (2016). Prioritization and burden analysis of rare variants in 208 candidate genes suggest they do not play a major role in CAKUT. Kidney Int.

[24] Ng SB, Bigham AW, Buckingham KJ (2010). Exome sequencing identifies MLL2 mutations as a cause of Kabuki syndrome. Nat Genet.

[25] Paulussen AD, Stegmann AP, Blok MJ (2011). MLL2 mutation spectrum in 45 patients with Kabuki syndrome. Hum Mutat.

[26] Sanna-Cherchi S, Khan K, Westland R (2017). Exome-wide Association Study Identifies GREB1L Mutations in Congenital Kidney Malformations. Am J Hum Genet.

[27] Petrovski S, Aggarwal V, Giordano JL (2019). Whole-exome sequencing in the evaluation of fetal structural anomalies: a prospective cohort study. Lancet.

[28] Hu P, Qiao F, Wang Y (2018). Clinical application of targeted next-generation sequencing in fetuses with congenital heart defect. Ultrasound Obstet Gynecol.

[29] van der Ven AT, Connaughton DM, Ityel H (2018). Whole-exome sequencing identifies causative mutations in families with congenital anomalies of the kidney and urinary tract. J Am Soc Nephrol.

[30] Kosfeld A, Brand F, Weiss AC (2017). Mutations in the leukemia inhibitory factor receptor (LIFR) gene and Lifr deficiency cause urinary tract malformations. Hum Mol Genet.

[31] Acién P, Acién M (2021). Renal agenesis, associated genital malformations, and responsible genes. Fertil Steril.

[32] Connaughton DM, Kennedy C, Shril S (2019). Monogenic causes of chronic kidney disease in adults. Kidney Int.

[33] Morcel K, Watrin T, Pasquier L (2011). Utero-vaginal aplasia (Mayer-Rokitansky-Kuster-Hauser syndrome) associated with deletions in known DiGeorge or DiGeorge-like loci. Orphanet J Rare Dis.

[34] Ledig S, Tewes AC, Hucke J (2018). Array-comparative genomic hybridization analysis in patients with Mullerian fusion anomalies. Clin Genet.

[35] Labastie MC, Catala M, Gregoire JM, Peault B (1995). The GATA-3 gene is expressed during human kidney embryogenesis. Kidney Int.

[36] Hwang DY, Dworschak GC, Kohl S (2014). Mutations in 12 known dominant disease-causing genes clarify many congenital anomalies of the kidney and urinary tract. Kidney Int.

[37] Van Esch H, Groenen P, Nesbit MA (2000). GATA3 haplo-insufficiency causes human HDR syndrome. Nature.

[38] Hernández AM, Villamar M, Roselló L, Moreno-Pelayo MA, Moreno F, Del Castillo I (2007). Novel mutation in the gene encoding the GATA3 transcription factor in a Spanish familial case of hypoparathyroidism, deafness, and renal dysplasia (HDR) syndrome with female genital tract malformations. Am J Med Genet A.

