# Supplementary material for: Variants in genes related to development of the urinary system are associated with Mayer–Rokitansky–Küster–Hauser syndrome
Source: Hum Genomics. 2022 Mar 31;16:10. doi: 10.1186/s40246-022-00385-0 (PMC8969342; doi:10.1186/s40246-022-00385-0)
Supplement: Supplementary file 1 — Additional file 1: Table 1. PCR Primers used in Sanger sequencing validation. [file 40246_2022_385_MOESM1_ESM.docx]

Supplementary Table 1. PCR Primers used in Sanger sequencing validation.

| **Genes and variants** | **Forward Primer** | **Reversed Primer** |
| --- | --- | --- |
| *TBC1D1*: c.2553delC | GGGTGACAAAGCCAGACTCTGT | TGCAGCCTTAATGAACACCCT |
| *TBC1D1*: c.1069G>C | CAGTGAGCTGAGATTGTGCCAT | TTGTAAAATGCTAGACCAGAAGGTG |
| *DLG5*: c.418C>T | GGTGGTTACTGGGGGTTGTTG | AGACGGCGCAATTACTACCTCT |
| *HOXD3*: c.575C>G | GGGCAGAGTGAACTGGATCTCA | TGGCCTTCTGGTCCTTCTTGTA |
| *GLI3*: c.895C>G | TAAACAGAGCTCCCCCGAGG | GGTGCAAACAAGTGCTGACATTA |
| *HIRA*: c.845A>G | GCAAGCTCCAGAAATGGAGAGT | ACCCCAGGTGCAGAATAGAAAT |
| *GATA3*: c.1178C>T | GTGCATTTCAGAGGCAGCAA | CAAACATAATTTTGCTTTCTGCC |
