# Supplementary figures and images for: Variants in genes related to development of the urinary system are associated with Mayer–Rokitansky–Küster–Hauser syndrome
Source: Hum Genomics. 2022 Mar 31;16:10. doi: 10.1186/s40246-022-00385-0 (PMC8969342; doi:10.1186/s40246-022-00385-0)

A

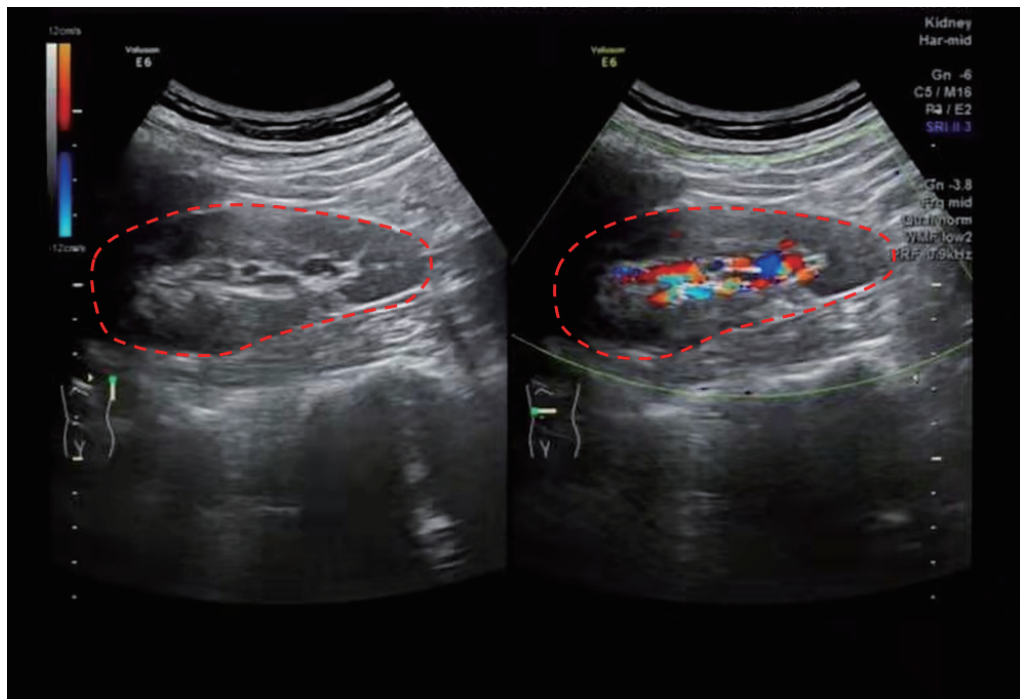

B

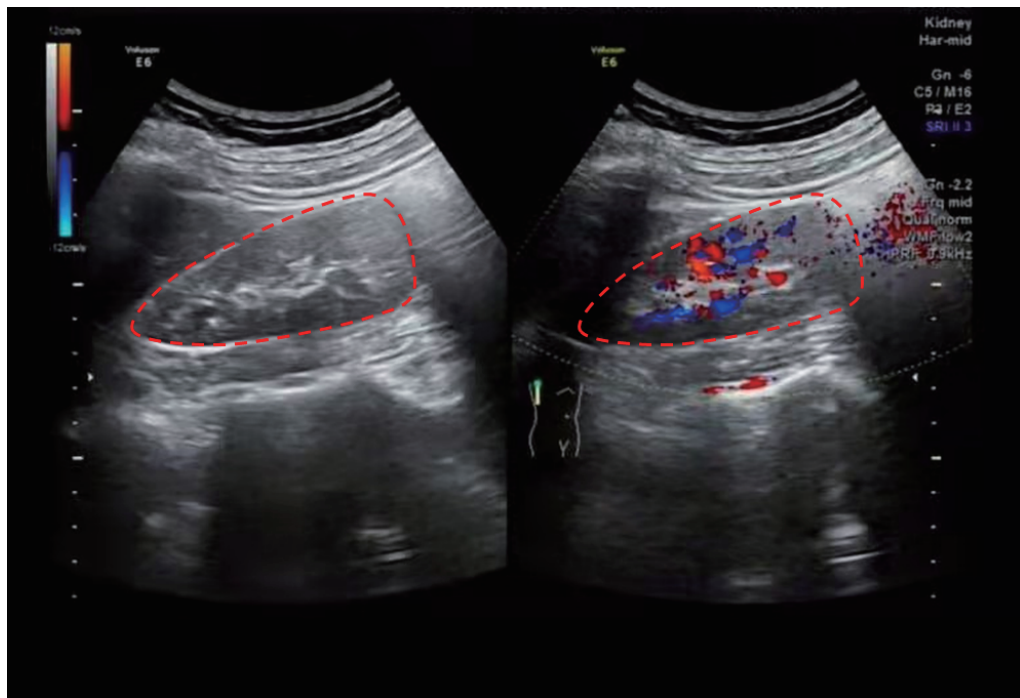

Supplement: Supplementary file 2 — Additional file 2: Fig. 1 Urinary ultrasound images of patient Fc-M-3. The left renal region (A) and right renal region (B) are normal. [file 40246_2022_385_MOESM2_ESM.pdf]

A

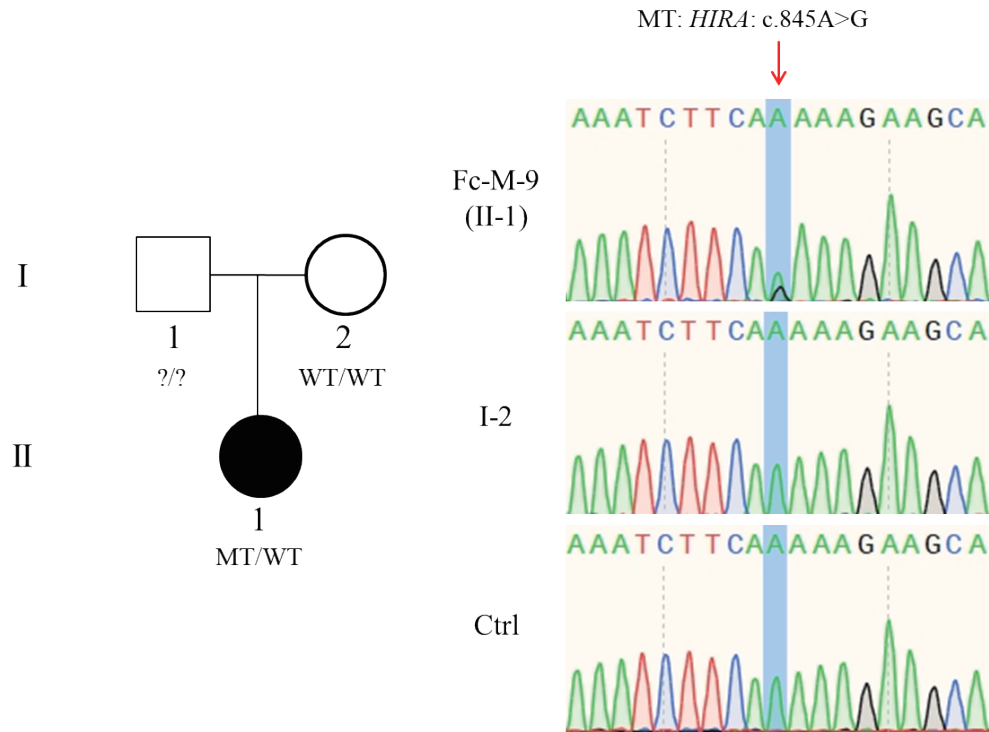

B

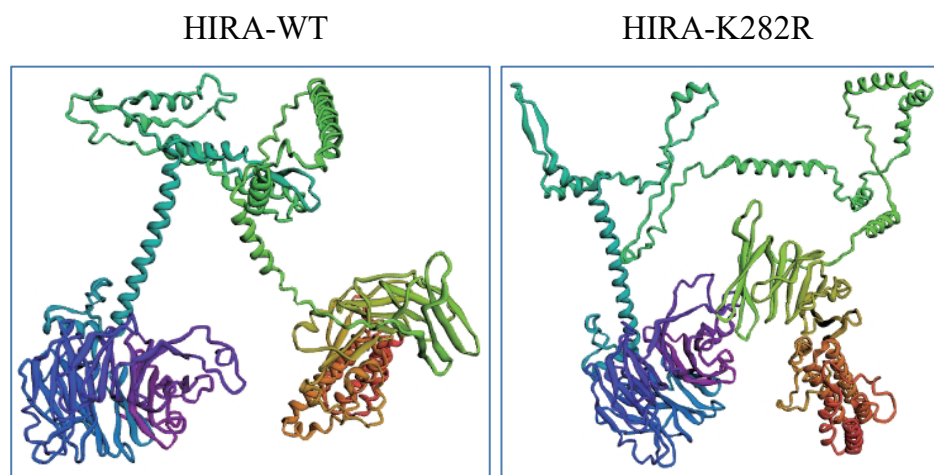

C

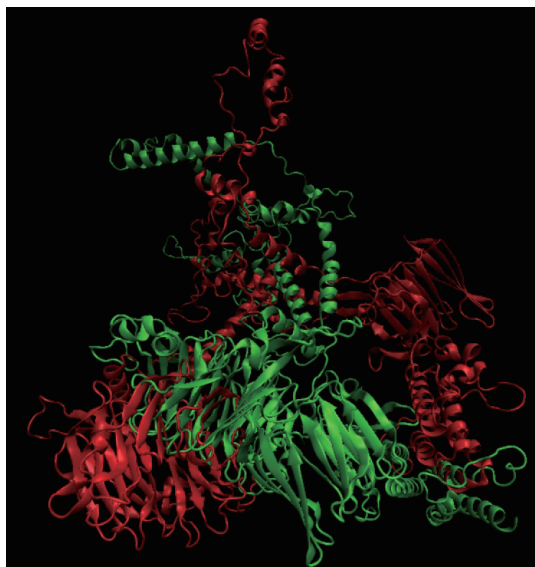

Supplement: Supplementary file 3 — Additional file 3: Fig. 2 HIRA variant is associated with MRKH syndrome. (A) Sanger sequencing validating the HIRA variant in patient Fc-M-9. The red arrow indicates the variant site c.845A>G. The patient’s mother did not harbor the variant. (B) The full-length wild-type (WT) HIRA protein and K282R mutant protein structures were predicted by RoseTTAFold. (C) The predicted protein structures for HIRA WT protein (green) and the K282R mutant protein (red) were aligned. The structure and conformation of K282R mutant protein have changed, so it is difficult for the K282R mutant protein to overlap with the WT protein. [file 40246_2022_385_MOESM3_ESM.pdf]

A

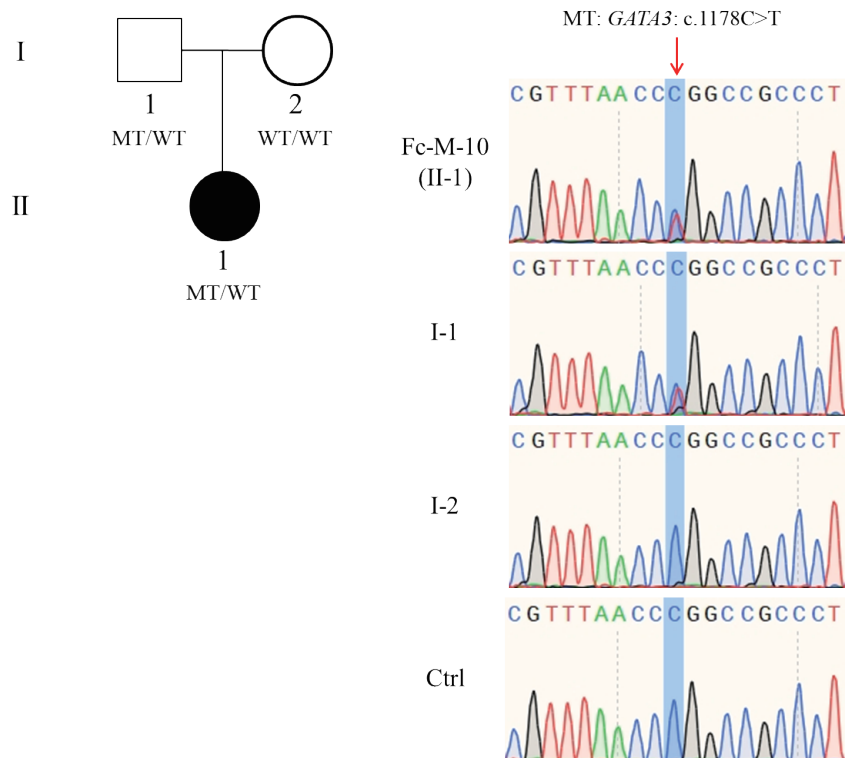

B

GATA3-WT

GATA3-P393L

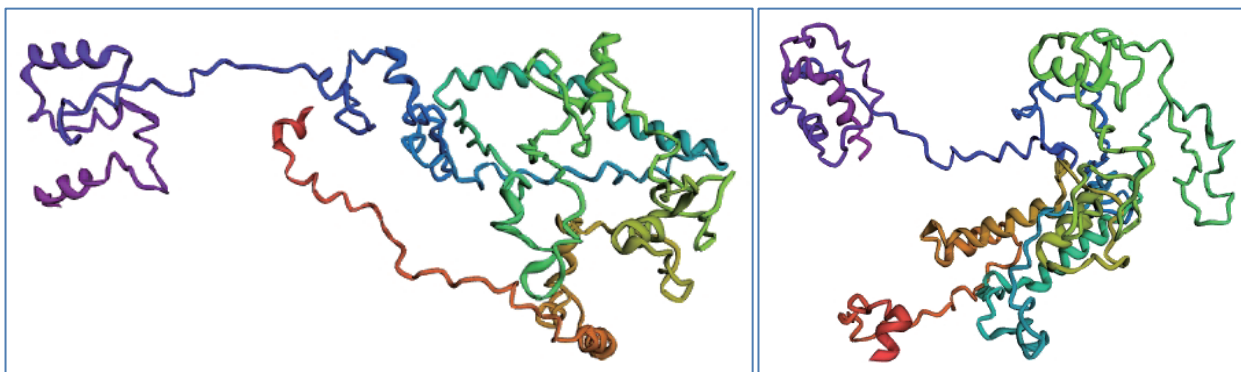

C

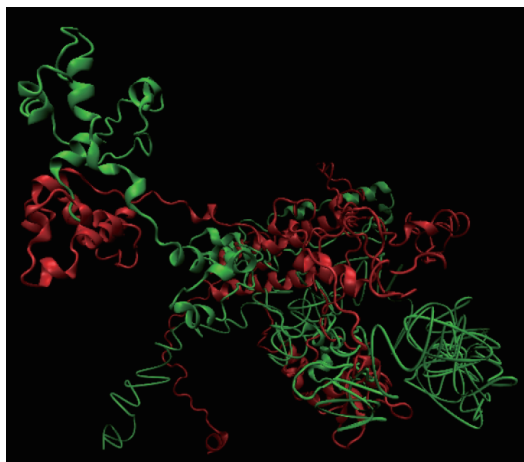

Supplement: Supplementary file 4 — Additional file 4: Fig. 3 GATA3 variant is associated with MRKH syndrome. (A) Sanger sequencing validating the GATA3 variant in patient Fc-M-10. The red arrow indicates the variant site c.1178C>T. (B) The full-length GATA3 wild-type (WT) protein and P393L mutant protein structures were predicted by RoseTTAFold. (C) The predicted protein structures for GATA3 WT protein (green) and the P393L mutant protein (red) were aligned. The predicted WT protein structure is more stretched, while the predicted P393L mutant protein structure is more compact. It is difficult for them to structurally overlap. [file 40246_2022_385_MOESM4_ESM.pdf]
